# Anxiety, self-efficacy, and their determinants in school students during the COVID-19 pandemic: a survey in Southeastern Iran

**DOI:** 10.1186/s12888-023-05252-3

**Published:** 2023-10-10

**Authors:** Limin Liu, Abdollah Dakkalirad, Mahlagha Dehghan, Allahyar Shahnavazi, Mahboobeh Maazallahi, Min Li, Mehri Kordi, Hojjat Farahmandnia, Mohammad Ali Zakeri

**Affiliations:** 1 Mental Health Education and Consultation Center, Jiaozuo Normal College, Jiaozuo, Henan 454000 China; 2 Tropical and communicable Disease Research Center, Iranshahr University of Medical Sciences, Iranshahr, Iran; 3 The Islamic Azad University, Tehran Medical Branch, Tehran, Iran; 4 Nursing Research Center, Kerman University of Medical Sciences, Kerman, Iran; 5 School of Nursing and Midwifery, Iranshahr University of Medical Science, Iranshahr, Iran; 6 Department of Critical Care Nursing, Kerman University of Medical Sciences, Kerman, Iran; 7 Department of Foreign Language and Economics, Jiaozuo Normal College, Jiaozuo, Henan 454000 China; 8 Nursing office, Iranshahr University of medical, Iranshahr, Iran; 9 Health in Disasters and Emergencies Research Center, Institute for Futures Studies in Health, Kerman University of Medical Sciences, Kerman, Iran; 10 Clinical Research Development Unit, Ali-Ibn Abi-Talib Hospital, Rafsanjan University of Medical Sciences, Rafsanjan, Iran; 11 Social Determinants of Health Research Center, Rafsanjan University of Medical Sciences, Rafsanjan, Iran

**Keywords:** COVID-19, Coronavirus, Anxiety, Self-efficacy, Students

## Abstract

**Background:**

The COVID-19 pandemic has been linked to various psychological disorders, including anxiety, particularly among the general public. It is crucial to monitor the mental health of school students, who are considered a vulnerable group in society, and assess their self-efficacy, as it can significantly affect their mental health. This study aimed to investigate the levels of anxiety and self-efficacy among school students during the COVID-19 pandemic.

**Methods:**

This cross-sectional study utilized convenience sampling to examine a sample of 335 school students from Sistan and Baluchestan province in southeastern Iran. An online questionnaire, distributed through social media platforms, was used to collect data. The questionnaire included demographic information, COVID-19 related items, the Multidimensional Anxiety Scale for Children-MASC, and the Self-Efficacy Questionnaire for Children-SEQ-C. Data analysis was conducted using IBM SPSS version 24, with a significance level set at < 0.05.

**Results:**

The study revealed a significant negative correlation between children’s anxiety and self-efficacy (r = -0.23, P < 0.001). Several factors were identified as predictors of anxiety, including low self-efficacy (β = -0.29, P < 0.001), female gender (β = 0.27, P < 0.001), concern about family members contracting COVID-19 (β = 0.18, P < 0.001), persistent advice of others to adopt preventive measures (β = 0.14, P < 0.005), level of education (β = -0.12, P < 0.017), and perceived risk of COVID-19 infection (β = -0.11, P < 0.030). These variables collectively accounted for 17% of the variance in anxiety.

**Conclusion:**

The findings of the study highlight the importance of addressing the mental health of school students, specifically their anxiety levels, during epidemics. The results also indicate that enhancing self-efficacy among students during a pandemic could be a beneficial strategy for promoting their mental health.

## Introduction

SARS-CoV-2, the virus responsible for COVID-19, was initially identified in Wuhan, China in late 2019 [1]. COVID-19 has emerged as a significant social stressor, posing a substantial threat to people’s health and safety [2, 3]. The fear of contracting the disease, feelings of hopelessness and mortality, the uncertain nature of the virus, and the extensive measures implemented to contain its spread [4], can have a profound impact on various aspects of life and disrupt mental health, in addition to physical health issues [5]. Throughout the COVID-19 pandemic, a wide range of mental disorders, including panic attacks, psychotic disorders, delusions, depression, anxiety, and even suicide, have been reported [6, 7]. Fear and anxiety are common responses to unfamiliar circumstances like the COVID-19 pandemic and may contribute to the high prevalence of psychological disorders during epidemics [8]. Anxiety is characterized by inner turmoil, feelings of distress, and anxiety about oneself or others in the face of potential threats [9].

Anxiety can play a crucial role in early detection of health issues and the adoption of health-promoting behaviors during an infectious disease outbreak [10]. An umbrella review conducted by Hossain et al. (2022) revealed a high prevalence of anxiety, depression, suicidal behavior, stress-related disorders, and other mental health problems among children and adolescents during the COVID-19 pandemic [11]. The findings of Oliveira et al. (2022) demonstrated significant variation in the proportion of children and adolescents experiencing mental health effects during the pandemic across different countries, with anxiety ranging from 17.6 to 43.7%, depression from 6.3 to 71.5%, and stress from 7 to 25% [12]. Studies conducted by Xie et al. (2020) in China and Alves et al. (2020) in California identified signs of anxiety in children aged 9 to 15 years during the COVID-19 pandemic [13, 14]. Mangolian Shahrbabaki et al. in Iran found that factors such as culture, education level, and birth rate could influence anxiety levels in girls aged 7–11 years old [15]. Ariapooran and Khezeli reported significant symptoms of anxiety disorders (ADs) among Iranian adolescents (aged 12 to 18 years) with hearing loss during the COVID-19 pandemic [16]. Therefore, recognizing and assessing anxiety can have a significant impact on the success or failure of general health strategies employed to manage pandemic diseases [10].

Due to the widespread economic and social disruptions caused by the pandemic, school students remain at significant risk for negative outcomes [17]. The consequences of COVID-19 on children’s health, safety, and well-being are substantial [18]. It is important not to underestimate the impact of changes in children’s daily lives, as the health and emotional states of other family members can affect them, and the negative effects of the environment can greatly influence their health. The COVID-19 pandemic has resulted in isolation and restrictions that significantly disrupt children’s lives [19].

Protective strategies such as mindfulness, flexibility, self-efficacy, and coping mechanisms are crucial in addressing psychosocial issues, as they can mitigate the severity of the disease in individuals [20]. Self-efficacy, which refers to an individual’s belief in their ability to succeed in specific situations or accomplish tasks, has been found to be effective in reducing stress among individuals facing challenging life events [21]. Individuals’ beliefs in their ability to control their behavior directly affect their tolerance threshold and their adherence to precautions, making it an important factor in promoting motivation and health-related behaviors [22]. Research indicates that self-efficacy plays a positive role in maintaining optimism and mental health during the COVID-19 pandemic [23, 24]. Conversely, individuals experiencing anxiety often exhibit fearful and avoidant behaviors that interfere with their daily functioning. In social cognitive theory, perceived efficacy plays a key role in triggering anxiety in students [25]. Lewis et al. (2020) demonstrated that self-efficacy played an important role in reducing anxiety among children aged 8–17 years in Maryland [26].

Several studies have highlighted the significance of considering the circumstances of families and children when implementing public health measures [17, 27]. Considering that the challenges associated with the COVID-19 pandemic can affect the well-being of school students, the objective of this study was to examine anxiety, self-efficacy, and the factors influencing these variables among school students during the COVID-19 pandemic.

## Methods

### Study design and setting

During the COVID-19 outbreak, a cross-sectional study was conducted to examine anxiety, self-efficacy, and their determinants among school students in Sistan and Baluchestan Province, located in southern Iran. Sistan and Baluchestan is the second largest province in Iran and shares borders with Pakistan and Afghanistan, with Zahedan as its capital. Data collection took place from July to October 2020.

### Sample size and sampling

An online questionnaire was developed and distributed through social networks, specifically WhatsApp. The sample consisted of all school students residing in Sistan and Baluchestan province, including elementary and junior high school students aged 8–18, living in both urban and rural areas. In order to participate, individuals needed to complete the survey in Farsi, have access to the internet, and be willing to complete the questionnaire. Incomplete questionnaires, where participants answered less than 90% of the questions, were excluded from the study, resulting in a non-response rate of 11.15%.

The target population for this study was school students at the time of data collection, with a total of 3000 individuals. The sample size was determined using the Cochran formula (α = 0.05, d = 0.05, Z = 1.94), resulting in a sample size of 341. Considering a 5% dropout probability, a total of 377 school students were selected for the study.

### Measurement

#### Demographic information

The demographic information collected from the participants encompassed various factors, including age, sex, education level, number of siblings, parents’ education level, birth rate, parents’ occupation, family income, parental employment in healthcare, exposure to COVID-19, preventive measures against COVID-19, concern about COVID-19 infection, presence of illness in the family, symptoms of COVID-19, and sources of information about COVID-19.

#### Multidimensional anxiety scale for children (MASC)

March et al. (1997) developed the 39-item self-report Multidimensional Anxiety Scale for Children (MASC) to assess anxiety and its symptoms in children aged 8–19 years old. The MASC consists of subscales, including social anxiety (9 items), separation anxiety (9 items), harm avoidance (9 items), and physical symptoms (12 items). Each item is rated on a four-point Likert scale ranging from zero to three (never, rarely, sometimes, and always), resulting in a total score range of 0 to 117 [28]. The internal consistency of the MASC was examined in a study by Kingery et al., yielding an alpha coefficient of 0.84 for the entire scale [29]. In Iran, the scale was validated by Karimi et al., who reported a Cronbach’s alpha coefficient of 0.79 for the overall scale [30]. In the present study, the Cronbach’s alpha coefficient for the Children’s Depression Inventory (CDI) was 0.86.

#### Self-efficacy questionnaire for children (SEQ-C)

In previous research, Muris utilized the Self-Efficacy Questionnaire for Children (SEQ-C), a 23-item scale, to evaluate the self-efficacy of children and adolescents across four domains: social (8 items), educational (8 items), emotional (7 items), and general. Each item is rated on a five-point Likert scale, ranging from 1 to 5, indicating the level of self-efficacy. Consequently, scores on the SEQ-C range from 23 to 115. The original version of the scale demonstrated a Cronbach’s alpha coefficient of 0.86 [31]. In an Iranian study conducted by Habibi et al. (2014), the psychometric properties of the scale were examined, confirming its construct validity through confirmatory factor analysis and its convergent validity through the Children Depression Inventory. The Cronbach’s alpha coefficients for the social, educational, emotional domains, and the overall scale were reported to be 0.73, 0.82, 0.76, and 0.85, respectively [32]. In the present study, the Cronbach’s alpha coefficients for the social, educational, emotional domains, and the overall scale of the SEQ-C were found to be 0.76, 0.89, 0.85, and 0.90, respectively.

#### Data collection and analysis

In addition to face-to-face training, social networks were utilized in schools for monitoring and communication purposes. The research team collaborated with school principals to distribute the questionnaire through these online social networks. Following the acquisition of necessary permissions, an online questionnaire was developed by the research team and computer experts, incorporating demographic information, COVID-related items, the MASC, and the SEQ-C. The efficiency and responsiveness of this online questionnaire were tested on a sample of 30 school students. A total of 380 questionnaires were collected, with 45 incomplete questionnaires being excluded, resulting in an effective response rate of 88.15%.

Subsequently, the collected data were analyzed using SPSS 22 (IBM Corp., Armonk, N.Y., USA). Descriptive statistics such as frequency, percentage, mean, and standard deviation were employed to describe the data. Pearson correlation coefficients were used to examine the relationships between the quantitative variables in the study. The independent t-test and ANOVA test were employed to assess the differences in anxiety and self-efficacy based on the qualitative variables in the study. Furthermore, multivariate linear regression was conducted to identify the determinants of anxiety and self-efficacy. The assumptions of the multiple regression model, including a linear relationship between variables, a normal distribution with zero mean, and constant variance, were considered. The significance level of 0.05 was used for all statistical analyses.

#### Ethical considerations

This research has a code of ethics No. IR.IRSHUMS.REC.1399.008 from Iranshahr University of Medical Sciences. Participants were informed that their responses would be used solely for research purposes and that their anonymity and confidentiality would be maintained. They were provided with a clear explanation of the study’s objectives and the voluntary nature of their participation. Participants explicitly agreed to these terms at the beginning of the online questionnaire.

## Results

Table 1 displays the demographic characteristics of the school students. The mean age of the participants was 14.21 ± 2.04 years. The majority of the participants were female. Additionally, most of the students were in their first year of high school and reported having two siblings. The fathers of the students were predominantly self-employed, while the mothers were primarily housewives, unemployed, or retired.

**Table 1:** Demographic characteristics of the participants and their associations with self-efficacy (n = 335)

Variables	Frequency (Valid percent)	Anxiety	Statistical test / P value	Self-Efficacy	Statistical test / P value
		**Mean** ± **SD**		**Mean** ± **SD**	
**Gender**					
Male	81 (24.2)	46.74 ± 14.95	t = -3.70 (< 0.001)	78.11 ± 11.98	t = -1.08 (0.22)
Female	254 (75.8)	54.00 ± 15.51		80.11 ± 15.14	
**Level of education**					
Primary	29 (8.7)	52.44 ± 13.82	F = 1.67 (0.18)	81.06 ± 9.80	F = 3.02 (0.05)
First High School	206 (61.5)	53.36 ± 15.57		80.85 ± 14.24	
Secondary school	100 (29.9)	49.88 ± 16.24		76.67 ± 15.64	
**Sibling**					
0	7 (2.1)	52.85 ± 11.34	F = 0.67 (0.67)	80.42 ± 19.04	F = 0.99 (0.43)
1	41 (12.2)	54.36 ± 14.26		80.56 ± 14.81	
2	90 (26.9)	50.14 ± 17.35		79.84 ± 14.37	
3	83 (24.8)	51.36 ± 14.25		79.73 ± 13.65	
4	49 (14.6)	54.44 ± 16.59		75.77 ± 13.80	
5	40 (11.9)	53.90 ± 13.43		79.82 ± 14.96	
6 ≤	25 (7.5)	52.16 ± 18.67		83.96 ± 15.76	
**Father’s job**					
Employed	119 (35.5)	51.92 ± 16.02	F = 0.04 (0.95)	81.00 ± 14.17	1.38 (0.25)
Self employed	130 (38.8)	52.34 ± 13.89		79.69 ± 14.32	
Unemployed – retired	86 (25.7)	52.54 ± 17.74		77.61 ± 14.94	
**Mother’s job**					
Employed	84 (25.1)	49.60 ± 15.38	F = 1.60 (0.20)	83.36 ± 12.87	F = 3.89 (0.021)
Self employed	43 (12.8)	53.27 ± 15.14		77.62 ± 17.01	
Housewife - unemployed – retired	208 (62.1)	53.10 ± 15.84		78.52 ± 14.28	
**Income of family** **(dollars)**†					
< 20	80 (23.9)	54.31 ± 18.04	F = 1.15 (0.32)	78.28 ± 16.76	F = 1.22 (0.30)
20–60	90 (26.9)	51.46 ± 15.50		79.91 ± 13.26	
60–100	80 (23.9)	53.27 ± 14.83		78.20 ± 13.00	
> 100	85 (25.3)	50.16 ± 14.07		81.92 ± 14.53	
**Father working in the health system**					
Yes	51 (15.2)	54.25 ± 14.38	t = 0.99 (0.32)	81.56 ± 13.07	1.04 (0.29)
No	284 (84.8)	51.88 ± 15.88		79.27 ± 14.67	
**Mother working in the health system**					
Yes	27 (8.1)	54.88 ± 15.59	t = 0.91 (0.36)	81.88 ± 13.51	0.84 (0.39)
No	308 (91.9)	52.01 ± 15.68		79.42 ± 14.53	

SD = Standard Deviation, t = Independent t test, F = Analysis of variance. † One dollar: 500 thousand rials

Out of the participants, the majority (306, 91.3%) did not contract the coronavirus, and they also did not have any relatives or friends who were infected (194, 57.9%). Additionally, 75.2% of the participants reported that preventive measures had a significant impact on preventing COVID-19, and over 60% of them believed they were not at risk of contracting the disease. Around 58% of the participants followed COVID-19 precautions such as regular hand washing and the use of disinfectants.

Regarding hand washing, the majority of participants (195, 58.2%) always adhered to the recommended precautions. Furthermore, a significant number of participants (128, 38.2%) expressed their primary concern about the possibility of their families being infected with COVID-19. More than 40% of the participants stated that they relied on social media as their main source of information about the pandemic (Table 2).

**Table 2:** The participants’ responses to COVID-related questions and their associations with self-efficacy (n = 335)

Variables	Frequency (Valid percent)	Anxiety	Statistical test / P value	Self-Efficacy	Statistical test / P value
		**Mean** ± **SD**		**Mean** ± **SD**	
**Infected with the coronavirus**					
Yes	29 (8.7)	52.24 ± 18.77	t = -0.002 (0.99)	76.89 ± 13.51	t = -1.06 (0.28)
No	306 (91.3)	52.24 ± 15.38		79.88 ± 14.52	
**Relatives/ friends infected with the coronavirus**					
Yes	194 (57.9)	53.16 ± 16.61	t = 1.25 (0.20)	78.94 ± 14.48	t = -1.00 (0.31)
No	141 (42.1)	50.98 ± 14.22		80.56 ± 14.40	
**Being at risk of coronavirus infection**					
Yes	130 (38.8)	53.86 ± 16.30	t = 1.50 (0.13)	77.15 ± 13.85	t = -2.51 (0.012)
No	205 (61.2)	51.22 ± 15.20		81.19 ± 14.63	
**Effectiveness of precautionary measures**					
Low	16 (4.8)	54.18 ± 24.51	F = 0.18 (0.83)	67.18 ± 15.24	F = 6.84 (0.001)
Medium	67 (20.0)	52.73 ± 14.63		78.86 ± 14.49	
Much	252 (75.2)	51.99 ± 15.30		80.61 ± 14.06	
**Precautions to prevent coronavirus infection**					
Seldom / sometimes	40 (11.9)	52.75 ± 19.76	F = 0.14 (0.86)	72.72 ± 13.78	F = 8.77 (< 0.001)
Most of the time	100 (29.9)	51.56 ± 15.24		77.59 ± 14.64	
Always	195 (58.2)	52.49 ± 15.01		82.08 ± 13.91	
**Adherence to hand washing and disinfection**					
Seldom / sometimes	42 (12.5)	51.69 ± 17.34	F = 0.08 (0.92)	70.54 ± 14.25	F = 15.69 (< 0.001)
Most of the time	98 (29.3)	52.73 ± 14.94		77.18 ± 13.57	
Always	195 (58.2)	52.12 ± 15.72		82.81 ± 13.90	
**Advising others to take preventative measures**					
Seldom / sometimes	89 (26.6)	50.32 ± 16.16	F = 1.64 (0.19)	73.58 ± 15.42	
Most of the time	109 (32.5)	51.59 ± 15.29		80.49 ± 14.09	F = 12.17 (< 0.001)
Always	137 (40.9)	54.01 ± 15.56		82.86 ± 12.87	
**The most important concern about the coronavirus**					
My family getting sick	128 (38.2)	55.25 ± 14.91	F = 3.90 (0.021)	80.50 ± 13.35	F = 0.77 (0.46)
Death	91 (27.2)	50.72 ± 14.82		80.08 ± 14.63	
Others	116 (34.6)	50.12 ± 16.71		78.29 ± 15.45	
**How to get information about the coronavirus disease**					
Medical staff	52 (15.5)	54.86 ± 16.23	F = 0.91 (0.43)	79.71 ± 13.71	
Internet	81 (24.2)	50.32 ± 14.77		79.49 ± 14.56	F = 0.03 (0.99)
Social Networks	155 (46.3)	52.50 ± 15.33		79.49 ± 13.96	
Others	47 (14.0)	51.80 ± 17.57		80.19 ± 16.90	

SD = Standard Deviation, t = Independent t test, F = Analysis of variance

The mean anxiety score among school students was 52.24 ± 15.67, which was lower than the midpoint of the questionnaire (score = 58.5). Among the MASC subscales, harm avoidance had the highest score, while separation anxiety had the lowest score. On the other hand, the mean self-efficacy score was 79.62 ± 14.44, which was higher than the midpoint of the questionnaire (score = 57.5). Among the self-efficacy subscales, academic self-efficacy had the highest score, while emotional self-efficacy had the lowest score (Table 3).

**Table 3:** Distribution of the anxiety and self-efficacy in school students (n = 335)

Variable	Mean	SD	Min	Max
Social Anxiety	11.13	5.99	0	27
Separation Anxiety	10.59	4.75	0	26
Harm Avoidance	18.78	4.51	2	27
Physical Symptoms	11.80	6.93	2	31
Anxiety	52.24	15.67	9	98
Social	27.20	5.52	11	40
Academic	30.30	6.21	9	40
Emotional	22.11	5.91	7	35
Self-Efficacy	79.62	14.44	36	114

A significant negative correlation was found between anxiety and self-efficacy among school students (r = -0.23; P < 0.001). However, only the separation anxiety subscale of MASC did not show a significant relationship with self-efficacy (P = 0.18). Additionally, academic self-efficacy was the only subscale of SEQ-C that did not have a significant relationship with anxiety (P = 0.08) (Table 4).

**Table 4:** Correlation between the anxiety and self-efficacy of school students (n = 335)

Variable	1	2	3	4	5	6	7	8
1. Social Anxiety	1							
2. Separation Anxiety	0.45 (< 0.001)	1						
3. Harm Avoidance	0.10 (0.47)	0.44 (< 0.001)	1					
4. Physical Symptoms	0.57 (< 0.001)	0.30 (< 0.001)	0.01 (0.81)	1				
**5. Anxiety**	0.79 (< 0.001)	0.74 (< 0.001)	0.47 (< 0.001)	0.74 (< 0.001)	1			
6. Social	-0.38 (< 0.001)	-0.21 (< 0.001)	0.12 (0.02)	-0.25 (< 0.001)	-0.27 (< 0.001)	1		
7. Academic	-0.27 (< 0.001)	0.08 (0.14)	0.33 (< 0.001)	-0.26 (< 0.001)	-0.09 (0.08)	0.46 (< 0.001)	1	
8. Emotional	-0.32 (< 0.001)	-0.06 (0.25)	0.21 (< 0.001)	-0.31 (< 0.001)	-0.21 (< 0.001)	0.48 (< 0.001)	0.55 (< 0.001)	1
**9. Self-Efficacy**	-0.39 (< 0.001)	-0.07 (0.18)	0.27 (< 0.001)	-0.33 (< 0.001)	**-0.23 (< 0.001)**	0.78 (< 0.001)	0.83 (< 0.001)	0.83 (< 0.001)

Data were presented as Pearson’s correlation coefficient

The bivariate analysis revealed that anxiety was significantly associated with sex and the primary concern about the coronavirus. Similarly, self-efficacy showed significant associations with mothers’ occupation, the effectiveness of precautionary measures, perceived risk of coronavirus infection, adherence to preventive measures such as hand washing and disinfectant use, and advice of others to take precautions (Tables 1 and 2).

Multiple regression models using the backward method were conducted to examine how demographic variables could predict anxiety among school students. As presented in Table 5, the predictors of anxiety included low self-efficacy, female gender, concern about family members contracting the coronavirus, persistent advice of others to take preventive measures, level of education, and perceived risk of coronavirus infection. These predictors accounted for 17% of the variance in anxiety (p < 0.001). Conversely, low anxiety, concern about family members contracting the coronavirus, older age, female gender, and persistent advice of others to take preventive measures accounted for 16% of the variance in self-efficacy (p < 0.001).

**Table 5:** Multiple regression analysis summary for underlying variables of the anxiety and Self-efficacy of school students (n = 335)

Variable		B	SE‡	β	t	P	95% Confidence interval for B	R[2]
**Anxiety**	Constant	60.95	6.8		8.87	< 0.001	47.44 _ 74.47	%17
	Self-efficacy	-0.32	0.05	-0.29	-5.65	< 0.001	-0.43 _ -0.21	
	Gender	9.89	1.95	0.27	5.07	< 0.001	6.06 _ 13.73	
	The most important concern about the coronavirus	3.32	0.94	0.18	3.52	< 0.001	1.47 _ 5.17	
	Advice of others to adopt preventative measures	2.82	1.00	0.14	2.82	0.005	0.85 _ 4.79	
	Level of education	-3.25	1.35	-0.12	-2.40	0.017	-5.91 _ -0.58	
	Perceived risk of coronavirus infection	-3.78	1.73	-0.11	-2.17	0.030	-7.20 _ -0.36	
**Self-efficacy**	Constant	85.64	6.96		12.29	< 0.001	71.93 _ 99.35	%16
	Anxiety	-0.28	0.04	-0.31	-5.88	< 0.001	-0.38 _ -0.19	
	Advice of others to adopt preventative measures	4.69	0.90	0.26	5.18	< 0.001	2.91 _ 6.47	
	The most important concern about the coronavirus	2.04	0.87	0.12	2.33	0.020	0.32 _ 3.76	
	Age	-0.84	0.35	-0.11	-2.34	0.020	-1.54 _ -0.13	
	Gender	3.73	1.76	0.11	2.11	0.035	0.26 _ 7.21	

Data were presented as multiple regression analysis. ‡: Standard error; Only significant results were shown; CI, Confidence intervals for B

## Discussion

In the current study, the anxiety levels of school students were found to be lower than the average. This finding contrasts with the study conducted by Yue et al. in China (2020), where children’s anxiety levels were reported to be mild to moderate. Yue et al. suggested that restrictions such as lockdown during an epidemic could provide an opportunity for improved parent-child interactions, potentially alleviating psychological distress and anxiety in children [33].

On the other hand, Pnar Senkalfa et al. in Turkey (2020) discovered that adolescents aged 13–18 experienced high levels of anxiety. Factors such as facing a health-threatening risk for the first time and lacking previous exposure to stress were found to contribute to their fear and anxiety [34]. Similarly, Espada et al. (2021) conducted a study in Spain, Italy, and Portugal involving children aged 3–18 and found higher-than-average levels of anxiety. Spanish children whose parents were stressed due to strict quarantine measures exhibited higher levels of anxiety. The authors emphasized the need for measures to support families in coping with epidemic-related stress and to identify and provide support to anxious children promptly to safeguard their mental health [35].

Measures implemented to control the spread of communicable diseases, including quarantine, social distancing, and economic changes, can have adverse effects on children and adolescents [36]. These measures often result in the loss of various physical and extracurricular activities, including attending school, which are integral to their daily lives. Consequently, children and adolescents become more vulnerable to the negative impacts of these measures [37].

Although the present study found lower-than-average anxiety levels among school students, the exact reasons for this finding cannot be definitively explained. It is possible that the prolonged duration of the COVID-19 pandemic or the timing of the study may have influenced the results. Therefore, further research is necessary to gain a better understanding of this phenomenon.

In the current study, the rate of self-efficacy among participants was found to be higher than average. This finding aligns with the research conducted by Hussong et al. (2021) in the United States, who emphasized the importance of increasing children’s self-efficacy as a factor in effectively coping with COVID-19 [38]. Similarly, San Carlos in the Philippines (2020) discovered that students exhibited high levels of self-efficacy during the COVID-19 outbreak and recommended implementing policies to enhance self-efficacy for achieving desired outcomes [39].

Lim et al. (2021) also found a high level of self-efficacy among the general population in China, Italy, and Singapore during the COVID-19 outbreak. The majority of respondents demonstrated adequate knowledge of how to deal with the disease in the event of infection and how to protect themselves and their family members from COVID-19 [40]. Additionally, Mohsenipouya et al. in Iran (2021) found that patients referred to corona centers exhibited good knowledge and self-efficacy. They suggested that educational interventions should be implemented to maintain and promote self-efficacy during infectious disease outbreaks, as self-efficacy had a positive effect on increasing the sensitivity and perceived severity of COVID-19 preventive behaviors [41]. It is important to note that self-efficacy influences an individual’s motivation and encourages them to persist with certain behaviors [42].

The current study revealed a significant inverse relationship between school students’ anxiety and self-efficacy, indicating that as anxiety levels decreased, self-efficacy increased, and vice versa. This finding is consistent with the research conducted by Arora et al. in India (2021), which also found a significant negative association between anxiety and self-efficacy. Similarly, Hussong et al. (2021) discovered that an increase in symptoms of mental disorders associated with the onset of the COVID-19 pandemic decreased with higher levels of self-efficacy and problem-focused coping among children aged 6–11 years in the United States [38].

These findings underscore the importance of enhancing self-efficacy as an effective coping strategy to reduce feelings of helplessness, perceived stress, and mental disorders during pandemics [38, 43].

The researchers aimed to enhance students’ self-efficacy in order to help them effectively manage their anxiety amidst the pandemic. They identified that academic institutions needed to strengthen their strategies, particularly focusing on self-efficacy, to effectively handle infectious diseases [44]. A study conducted by Alemany-Arrebola et al. in Canada (2020) revealed that COVID-19-related stressors, such as imposed restrictions, illness, or the loss of loved ones, were associated with increased anxiety among students. It was observed that students with higher anxiety levels had lower self-efficacy levels. Consequently, the researchers concluded that it was crucial to monitor students’ psychological well-being and assess the impact of the pandemic on their academic performance [45].

Furthermore, Bandura et al. emphasized the relationship between self-efficacy and behavioral problems, highlighting the unique role of self-efficacy in maintaining negative emotional states [46]. This underscores the significance of fostering self-esteem and a sense of efficacy in adolescents during their academic and social interactions. As Bandura noted, a strong sense of efficacy contributes to human achievement and personal well-being in various ways [47], ultimately reducing anxiety levels.

The current study identified several factors that influence the anxiety and self-efficacy of school students, including female gender, concerns about family members contracting coronavirus, and the tendency to advise others on preventive measures. These findings are consistent with the research conducted by Gao et al., which revealed that girls were more susceptible to mental health problems, anxiety, and depression, and were more sensitive to stressful situations compared to boys [48]. Similarly, Alemany-Arrebola et al. in Canada (2020) found that boys exhibited higher levels of self-efficacy than girls, which could be attributed to sociability processes and the persistence of gender stereotypes in the socio-cultural context of society [45].

Additionally, Garcia de Avila et al. (2020) discovered that factors such as social distancing without parents, living with more people in the household, and the education level of guardians were associated with higher anxiety scores [17]. Tatsiopoulou et al. (2022) also found that variables related to COVID-19, such as parental vaccine hesitancy and the loss of a loved one, significantly affected preschoolers’ anxiety levels [27]. These findings underscore the importance of considering various factors that influence children’s anxiety and self-efficacy, which should be taken into account by health managers. Therefore, it is crucial to implement public health strategies that strengthen families and provide support to parents and their children during critical situations [27]. Further studies in this area can help identify additional factors that contribute to these outcomes.

### Limitations

The study had limitations that should be considered when interpreting the results. These include the reliance on social media as the primary source of information, which may have excluded individuals without access to technology. Additionally, the study had a small sample size of self-reporting school students aged 8–18 years, which may limit the generalizability of the findings. It is possible that some students, particularly those in elementary school or rural areas, may not have had access to smartphones to complete the online questionnaire. Furthermore, younger students may have had difficulty answering the questionnaires accurately. More studies are needed to confirm the results. It is important to note that the investigated province is one of the most deprived in Iran, which may have made it challenging for participants to access mobile phones. As a result, only students with higher socio-economic status may have had access to the questionnaire. While parents were instructed not to answer the questions, the online nature of the survey requires caution when interpreting the results.

## Conclusion

The study findings indicated that school students experienced psychological disorders during the COVID-19 pandemic due to the stressful conditions and various disease control measures. However, the results also highlighted the importance of increased self-efficacy in mitigating these psychological disorders. Based on these findings, it is recommended that authorities implement interventions aimed at reducing anxiety and enhancing the self-efficacy of school students. These interventions can help improve their ability to cope with the stressors associated with the pandemic.

## Data Availability

The datasets used and/or analyzed during the current study are available from the corresponding author on reasonable request.
